# Supplementary material for: Longer and more frequent marine heatwaves over the past century
Source: Nat Commun. 2018 Apr 10;9:1324. doi: 10.1038/s41467-018-03732-9 (PMC5893591; doi:10.1038/s41467-018-03732-9)
Supplement: Supplementary file 1 — Supplementary Info File #1(PDF 4397 kb) [file 41467_2018_3732_MOESM1_ESM.pdf]

Longer and more frequent marine heatwaves over the past century

Supplementary Information

Eric C. J. Oliver[1,2,3*], Markus G. Donat[4,5], Michael T. Burrows[6], Pippa J. Moore[7], Dan A. Smale[8,9], Lisa V. Alexander[4,5], Jessica A. Benthuysen[10], Ming Feng[11], Alex Sen Gupta[4,5], Alistair J. Hobday[12], Neil J. Holbrook[2,13], Sarah E. Perkins-Kirkpatrick[4,5], Hillary A. Scannell[14,15], Sandra C. Straub[9], Thomas Wernberg[9]

1. Department of Oceanography, Dalhousie University, 1355 Oxford Street, Halifax, Nova Scotia B3H 4R2, Canada.
2. Institute for Marine and Antarctic Studies, University of Tasmania, 20 Castray Esplanade, Battery Point, Private Bag 129, Hobart, Tasmania 7001, Australia.
3. Australian Research Council Centre of Excellence for Climate System Science, University of Tasmania, Private Bag 129, Hobart, Tasmania 7001, Australia.
4. Climate Change Research Centre, University of New South Wales, Gate 11 Botany Street, Library Walk, Level 4, Matthews Building, Sydney, New South Wales 2052, Australia.
5. Australian Research Council Centre of Excellence for Climate System Science, University of New South Wales, Gate 11 Botany Street, Library Walk, Level 4, Matthews Building, Sydney, New South Wales 2052, Australia.
6. Department of Ecology, Scottish Association for Marine Science, Scottish Marine Institute, Oban, Argyll, PA37 1QA, Scotland, UK.
7. Institute of Biological, Environmental and Rural Sciences, Aberystwyth University, Aberystwyth SY23 3DA, UK.
8. Marine Biological Association of the United Kingdom, The Laboratory, Citadel Hill, Plymouth PL1 2PB, UK.
9. UWA Oceans Institute and School of Biological Sciences, The University of Western Australia, Crawley, Western Australia 6009, Australia.
10. Australian Institute of Marine Science, PMB 3, Townsville MC, Queensland 4810, Australia.
11. CSIRO Oceans and Atmosphere, Perth, Western Australia, Australia.

12. CSIRO Oceans and Atmosphere, Hobart, Tasmania 7000, Australia.
13. Australian Research Council Centre of Excellence for Climate Extremes, University of1 Tasmania, Private Bag 129, Hobart, Tasmania 7001, Australia
14. School of Oceanography, University of Washington, Seattle, Washington, USA.
15. NOAA Pacific Marine Environmental Laboratory, Seattle, Washington, USA.

* Corresponding author: eric.oliver@dal.ca

Supplementary Information

Supplementary Notes 1-2

Supplementary Table 1

Supplementary Figures 1-11

Supplementary Materials

Supplementary Note 1. Proxy performance for marine heatwave properties based on monthly SSTs

This section presents the performance of the proxy development for the daily station time series data and the monthly global SST data sets. We also discuss the properties of the proxy-based historical reconstruction of annual marine heatwave properties back to 1900.

The proxy models are described in the Methods, and here we present the results. The best proxy fit for each marine heatwave property was selected based on the correlation coefficient over the validation period (see results in Supplementary Table 1 and predicted time series in Supplementary Fig. 6, red lines). Marine heatwave frequency was best predicted from *NT* (the annual count of months above the 90th percentile) with a very good fit found for all six stations: correlation coefficients were between 0.51 and 0.94, all significant at the 1% level. Marine heatwave duration was best predicted from *TAX* (the annual maximum SST anomaly) with a good fit found for all but one station: correlation coefficients were between 0.32 and 0.99 (significant at least at the 10% level) at five of the six stations, with only Arendal providing a poor fit (0.09). The best model for intensity (predicted from *TAX*) showed a less consistent fit with correlation coefficients as high as 0.71 (Pacific Grove), as low as 0.23 (Arendal) and even negative at two stations (-0.39 at Port Erin and -0.51 at Race Rocks). In addition, the model uncertainty was relatively low for marine heatwave frequency and duration but was larger than the predicted variability for marine heatwave intensity (Supplementary Fig. 6, red shading). Therefore we do not consider proxies for intensity.

The best proxy models for the monthly global SST data sets were selected based on the global mean correlation coefficients of each model over the 1982-2016 period. The three best models selected in above again appeared here as either the best model (highest spatial-mean correlation coefficient) or within a correlation value of 0.05 of the best model. This was true for all data sets. The spatial distribution of the correlation coefficients for these selected models indicated a good fit ($p<0.05$) for marine heatwave frequency over the

vast majority of ocean locations (ranging from 88 to 97% across the five data sets; Supplementary Fig. 7A, E, I, M, Q), for marine heatwave duration over much of the ocean (41-72% across the data sets; Supplementary Fig. 7B, F, J, N, R), with the notable exception of the Southern Hemisphere higher latitudes, and for marine heatwave intensity over very little of the ocean surface (11-19%; Supplementary Fig. 7C, G ,K, O, S), except in the tropical eastern Pacific, the northern North Atlantic, and other small portions of the ocean. The probability distributions of correlation coefficients from all grid cells for each model (Supplementary Fig. 7D, H, L, P, T) showed that the model for frequency had a correlation coefficient >0.5 for most locations, the model for duration had correlation coefficients primarily in the 0.3-0.7 range, and the model for intensity had correlation coefficients <0.5 for most locations.

The spatial distributions of mean marine heatwave frequency and duration proxies (Supplementary Fig. 8, A and B) were generally consistent with the NOAA OI SST V2 results over the period 1982-2016 (Fig. 1, A and G). Over the common period of 1982-2016, the time series of globally averaged marine heatwave frequency was similar whether derived from the daily remotely-sensed SSTs (Fig. 5B, red line) or these monthly proxies (HadISST: r=0.85; ERSST: r=0.92; COBE: r=0.92; CERA20C: r=0.94; SODA: r=0.90; Data-set mean: r=0.93). This was also true for marine heatwave duration (Fig. 5D; HadISST: r=0.86; ERSST: r=0.90; COBE: r=0.92; CERA20C: r=0.88; SODA: r=0.78; Data-set mean: r=0.91). The proxies also match well regionally against the long station time series (Supplementary Fig. 9).

Observational errors were investigated using a proxy derived from HadSST3 data. For HadSST3, model error on the proxy for marine heatwave frequency, as represented by a 95% confidence interval, was on the order of 1 annual event (Supplementary Fig. 10A). Both the observational error (Supplementary Fig. 10B) and the total error (Supplementary Fig. 10C) were nearly identical to the model error indicating that the uncertainty on the frequency proxy was dominated by the covariance of the proxy time series themselves. Model error on the proxy for marine heatwave duration, again represented by a 95% confidence interval, was on the order of 5 days (Supplementary Fig. 10D). The observational error (Supplementary Fig. 10E) was slightly larger than the model error, by about 1 day

particularly in the pre-satellite era (before the 1980s) and therefore the total error (Supplementary Fig. 10F) was also slightly larger than the model error for this period, while nonetheless still dominated by the covariance term as above. The magnitude of the 95% confidence interval for both frequency and duration was smaller than the increase in those properties over the record length indicating the centennial-scale change was robust against both model and observational uncertainties.

Since sea surface temperature time series typically exhibit trends on decadal to multidecadal timescales our choice of climatology period defined what was considered a marine heatwave event. Our choice of a recent climatology period (1983-2012, same as used for the NOAA OI SST analysis), implied that all marine heatwave properties and trends are defined relative to what is considered a marine heatwave event in recent decades. As with all baseline-relative climate analyses, this means marine heatwave events in the early 20th century, when the overall climate was cooler, will be less frequent by definition and may lead to conservative estimates of change.

Supplementary Note 2. Excess trends in marine heatwave properties

The statistical model for determining excess trends in marine heatwave properties is described in the methods. Here we present results of the model fit and of excess trends at four example locations. The statistical model (Methods, Eq. 4) was fit to the daily NOAA OI SST data (1982-2015). The model parameters are shown in Supplementary Fig. 11. Regions with large values of the autoregressive time scale τ (Supplementary Fig. 11A) indicate these time series have longer memories (i.e., are more red noise-like) and included the equatorial eastern Pacific and the eddy-rich western boundary current extensions. Regions with large values of error variance σ_ε^2 (Supplementary Fig. 11B) were generally regions with large SST variance (e.g., western boundary current regions) but also included much the equatorial oceans and some mid-latitude oceans (e.g. the northeast and southeast Pacific).

This technique was demonstrated in detail at four locations where recent marine heatwaves have been observed (refs. [1–4]; see also introduction in the main text). We chose the following locations: the northern Mediterranean (43.5°N,9°E), off Western Australia

(29.5°S,112.5°E), the northwest Atlantic (43°N,67°W) and the northeast Pacific (44°N,144°W). For each location we fit the model to observed SSTs and then simulated trends in marine heatwave properties for a range of mean SST trends between 0 and 1 °C decade^{-1}, in 0.1 °C decade^{-1} steps. For each value of mean SST trend we simulated N_ε=1000 SST time series to generate an ensemble (Supplementary Fig. 3, grey lines) from which we calculated the ensemble mean (black line) and the 2.5th and 97.5th percentiles (blue and red lines respectively). Trends in marine heatwave properties always increased with a rising trend in mean SST (over the range considered) but this relationship was strongest for marine heatwave frequency. The observed marine heatwave property trends were compared against the simulated trends at the specific value of the observed mean SST trend (Supplementary Fig. 3, white circle), and if they were found to lie outside the domain bounded by the 2.5th and 97.5th percentiles, then we said that the property features an excess trend with p<0.05. Excess trends were found for marine heatwave intensity in the northeast Pacific and northern Mediterranean Sea. All other marine heatwave trends were within the 95% confidence interval of what was expected given only a rise in the mean SST.

We applied this technique globally using the NOAA OI SST data set, which for computational efficiency was restricted to every 4th pixel in latitude and longitude and an ensemble size N_ε=100. Regions with excess trends (p<0.05) in marine heatwave properties were shown globally in Fig. 3A-C. Analyses of higher order moments of the daily temperature distribution show that the excess trends can partly be explained by changes in SST variance and skewness (Fig. 3D-F). There was much overlap between regions of excess trends and regions of significant (p<0.05) trends in SST variance or skewness. For frequency, 61% of grid cells with excess trends corresponded to areas with significant trends in either the variance or skewness of SST. This proportion was 63% and 48% for intensity and duration respectively.

References

1. Sparnocchia, S., Schiano, M. E., Picco, P., Bozzano, R. & Cappelletti, A. The anomalous warming of summer 2003 in the surface layer of the Central Ligurian Sea (Western Mediterranean). *Ann. Geophys.* **24,** 443–452 (2006).

2. Pearce, A. F. & Feng, M. The rise and fall of the 'marine heat wave' off Western Australia during the summer of 2010/11. *J. Mar. Syst.* **111--112,** 139–156 (2013).

3. Chen, K., Gawarkiewicz, G. G., Lentz, S. J. & Bane, J. M. Diagnosing the warming of the Northeastern US Coastal Ocean in 2012: A linkage between the atmospheric jet stream variability and ocean response. *J. Geophys. Res. Ocean.* **119,** 218–227 (2014).

4. Bond, N. A., Cronin, M. F., Freeland, H. & Mantua, N. Causes and impacts of the 2014 warm anomaly in the NE Pacific. *Geophys. Res. Lett.* **42,** 1–7 (2015).

5. Corey, D. M., Dunlap, W. P. & Burke, M. J. Averaging correlations: Expected values and bias in combined Pearson rs and Fisher's z transformations. *J. Gen. Psychol.* **125,** 245–261 (1998).

Supplementary Table 1. Performance of marine heatwave proxies from century-long *in situ* records of ocean temperatures. The values shown are the correlation coefficient between annually-averaged marine heatwave properties and their prediction using proxies derived from monthly-mean temperatures (see Methods, Eq. 1). Correlations are shown for the model training period (1982 to the end of time series) and for the model validation period (beginning of time series to 1981). Roman, bold roman and bold italics indicate a value is significantly different from zero with 90%, 95% and 99% confidence, respectively, and italics indicates a value not significantly different from zero with 90% confidence.

	Training correlation (1982-)			*Validation correlation (-1981)*		
Station	*Frequency*	*Intensity*	*Duration*	*Frequency*	*Intensity*	*Duration*
Pacific Grove, USA	***0.83***	***0.73***	***0.77***	***0.89***	***0.71***	***0.61***
Scripps Pier, USA	***0.82***	***0.60***	***0.68***	***0.88***	***0.69***	0.36
Newport Beach, USA	***0.76***	**0.41**	**0.50**	***0.85***	**0.47**	*0.32*
Arendal, Norway	***0.86***	**0.53**	***0.64***	***0.58***	*0.23*	*0.09*
Port Erin, UK	***0.89***	**0.49**	***0.69***	***0.94***	*-0.39*	***0.70***
Race Rocks, Canada	***0.94***	*0.07*	***0.78***	***0.51***	*-0.51*	***0.99***

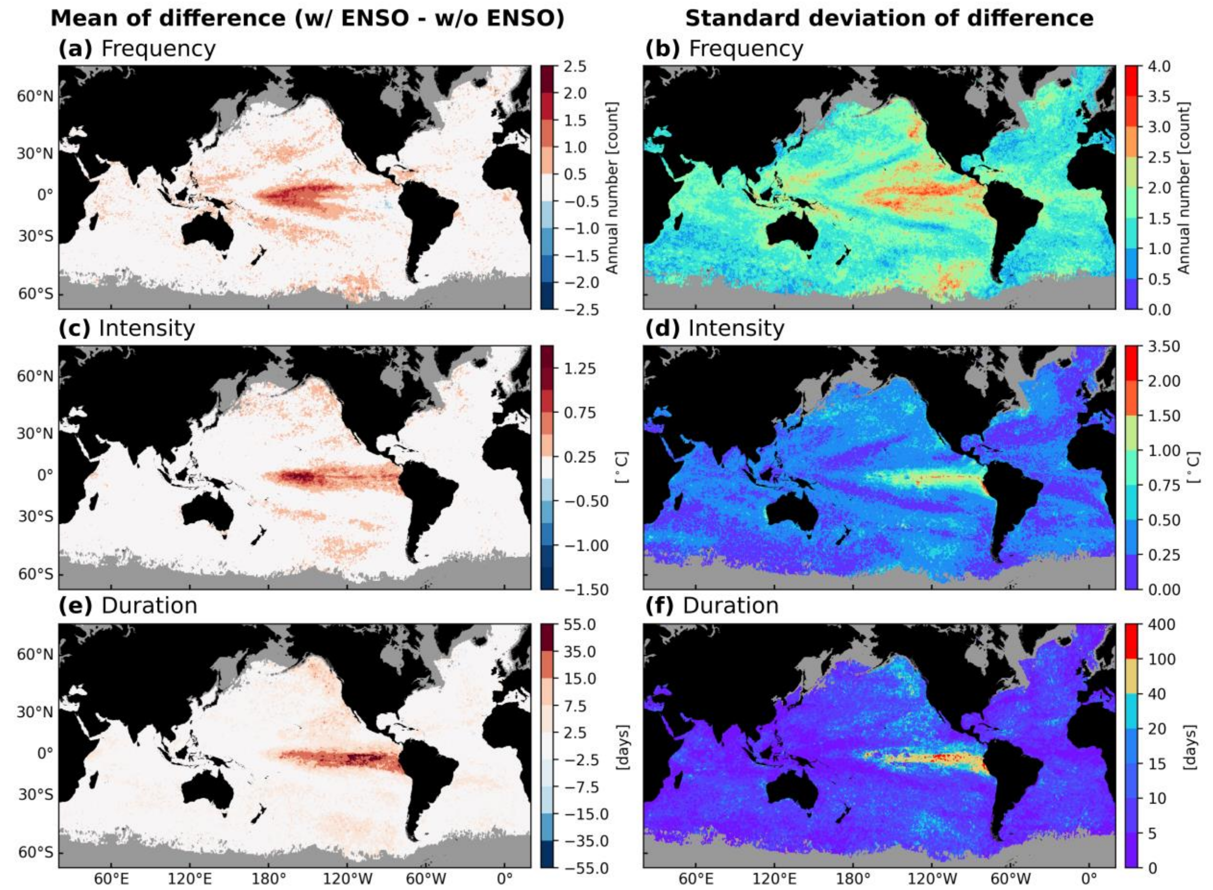

Supplementary Figure 1. Influence of ENSO on the mean and variability of MHW properties over 1982-2016. Shown are (A,C,E) the mean and (B,D,F) the standard deviation of the difference between the annual MHW (A,B) frequency, (C,D) intensity, and (E,F) duration before and after the removal of ENSO.

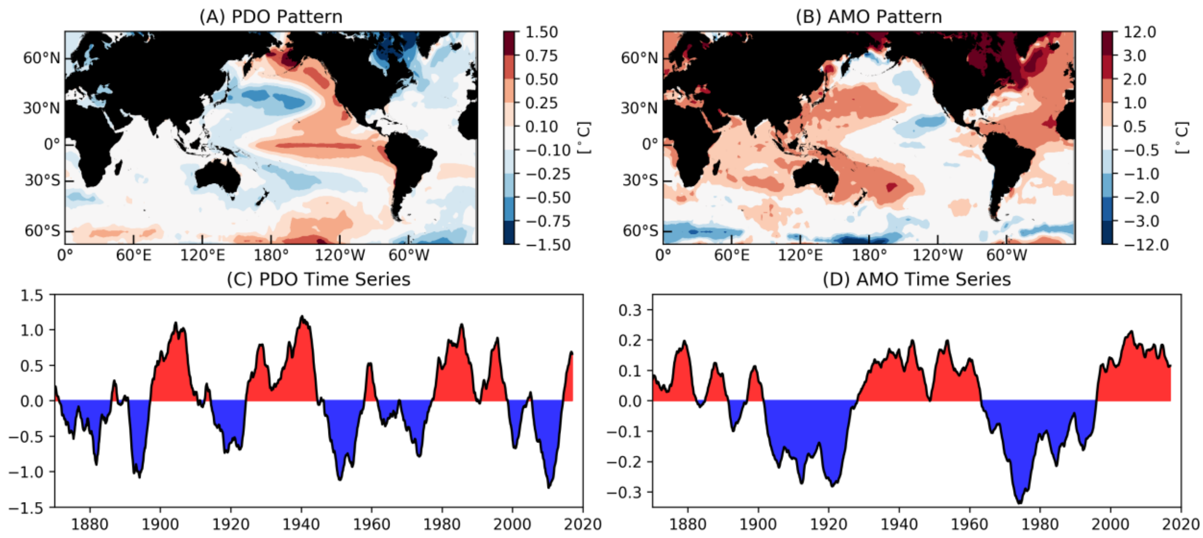

Supplementary Figure 2. Pacific Decadal Oscillation (PDO) and Atlantic Multidecadal Oscillation (AMO). Shown are (A,B) the spatial loading patterns for SST anomalies and (C,D) the associated timeseries for the PDO and the AMO. Data for the maps are taken from NCEP/NCAR Reanalysis 1.

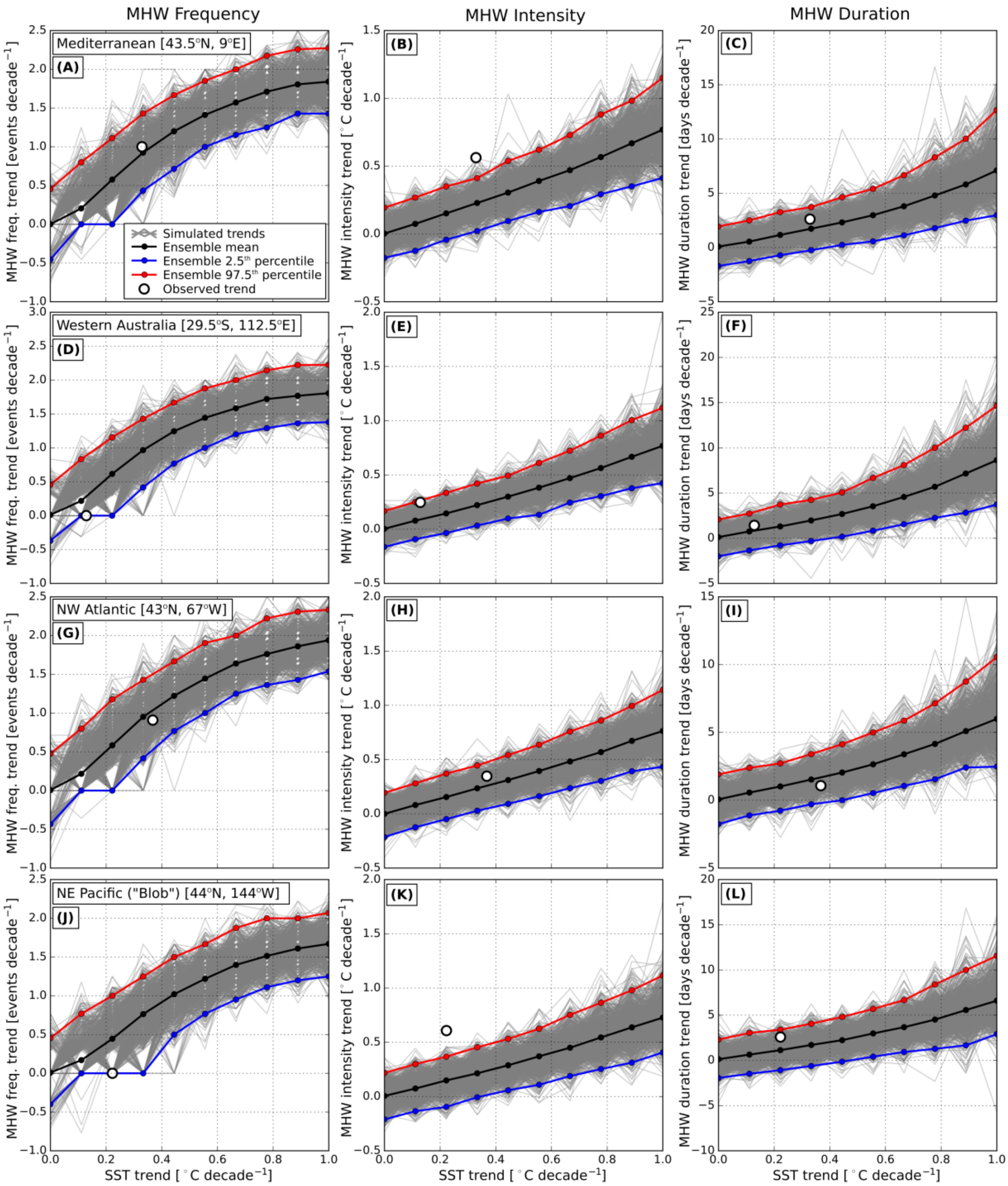

Supplementary Figure 3. Comparison of observed and simulated trends in marine heatwave properties at four locations. Shown are linear trends in (A,D,G,J) marine heatwave frequency, (B,E,H,K) marine heatwave intensity, and (C,F,I,L) marine heatwave duration as a function of linear trend in annual mean SST. Grey lines are results from a 1000-member ensemble of simulated SSTs using the stochastic climate model, assuming only linear

changes in the annual mean SST, presented in Supplementary Note 2. The ensemble mean and 2.5th and 97.5th percentiles are shown as black, blue, and red lines respectively. The white circles show the observed marine heatwave property trends, situated on the *x*-axis according to the observed trend in annual mean SST.

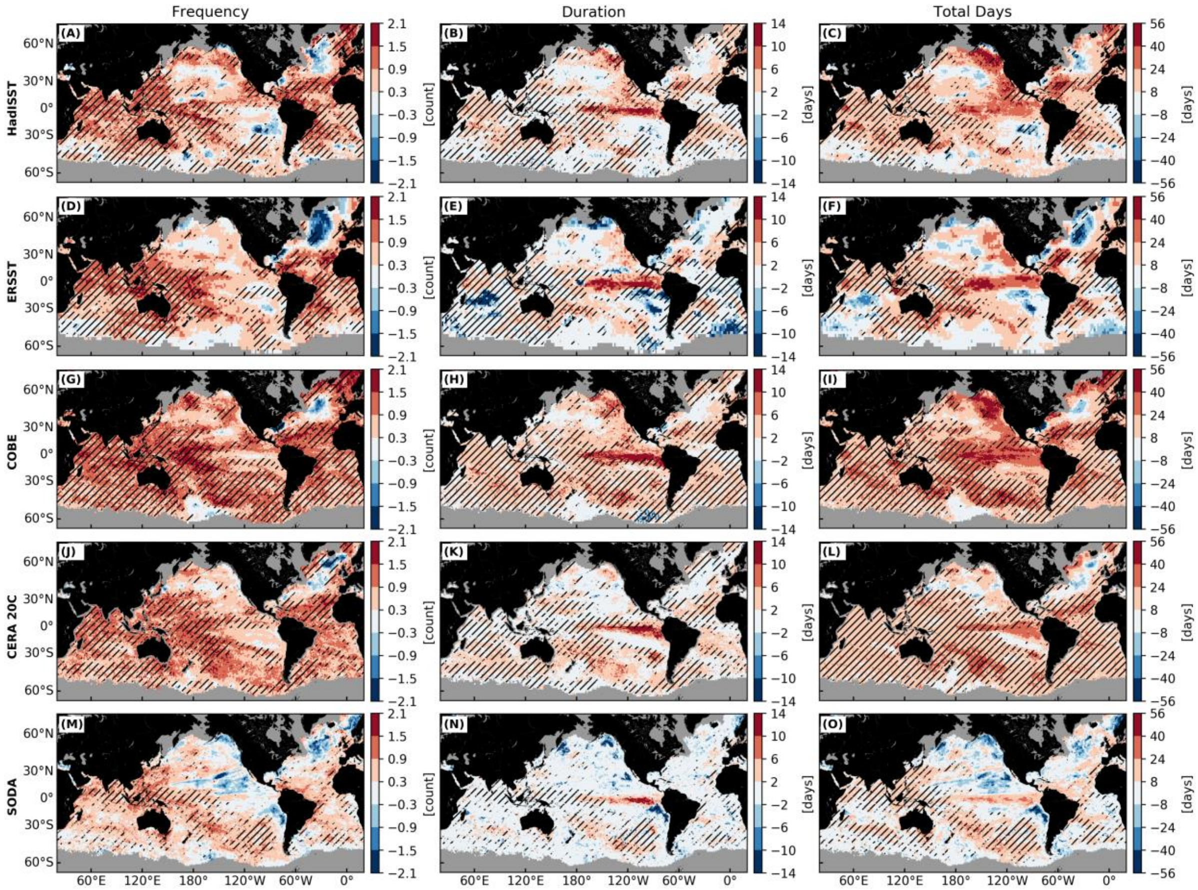

Supplementary Figure 4. **Centennial changes in marine heatwave (MHW) proxies derived from monthly gridded sea surface temperature (SST) datasets.** Difference between 1925-1954 and 1987-2016 of annual (A,D,G,J,M) MHW frequency, (B,E,H,K,N) MHW duration, and (C,F,I,L,O) total MHW days based on monthly proxies derived from HadISST, ERSST, COBE, CERA 20C and SODA datasets over 1900-2016. Hatching indicates the change is significantly different from zero at the 5% level and grey areas indicate no data.

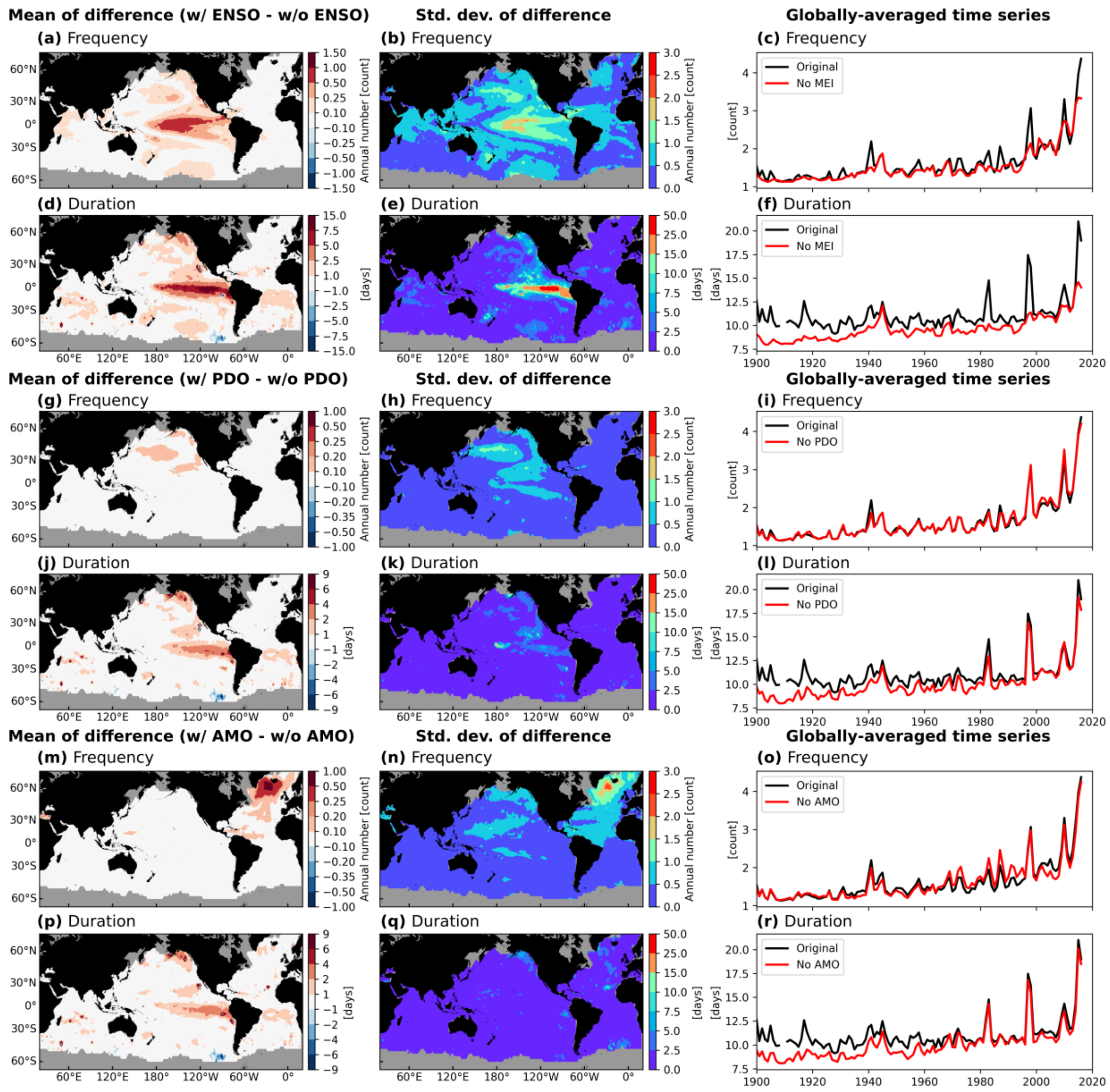

Supplementary Figure 5. Influence of ENSO, PDO and AMO on the mean, variability and globally-averaged time series of MHW properties over 1900-2016. Shown are (A,D,G,J,M,P) the mean and (B,E,H,K,N,Q) the standard deviation of the difference between the annual MHW frequency and duration before and after the removal of ENSO (A-F), PDO (G-L), and AMO (M-R). Also shown are (C,F,I,L,O,R) the corresponding globally-averaged time series with and without the signature of these modes of variability. The results presented have been averaged across the five proxy datasets.

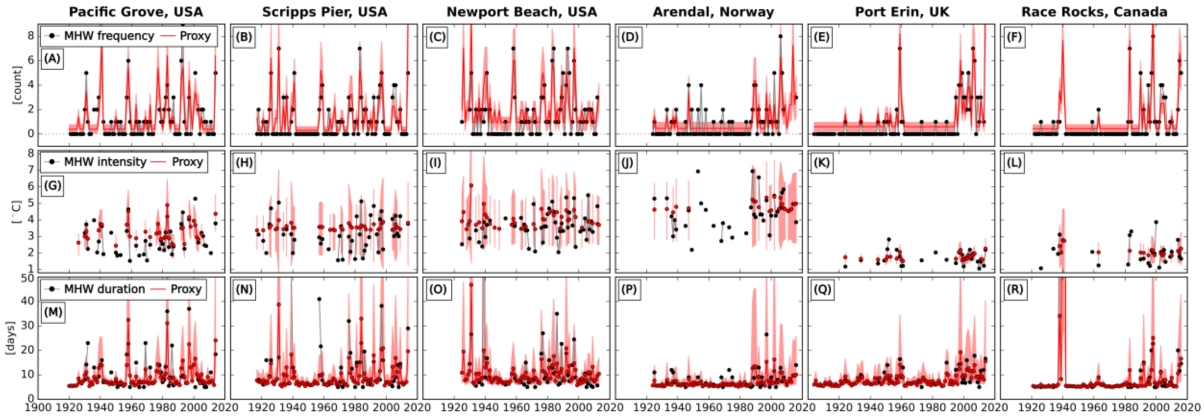

Supplementary Figure 6. Marine heatwave properties and their proxies at six sites with century-long daily *in situ* temperature records. Shown as black lines are (A-F) the number of marine heatwaves in each year, (G-L) the average annual marine heatwave intensity, (M-R) the average annual marine heatwave duration at Pacific Grove, Scripps Pier, Newport Beach (USA), Arendal (Norway), Port Erin (UK) and Race Rocks (Canada). Also shown in each panel is the corresponding proxy (red line) and 99% confidence interval (red shading) derived using monthly temperature data. See Table 1 and Supplementary Table 1 for more details on daily temperature records and the fit of the proxies to the marine heatwave metrics.

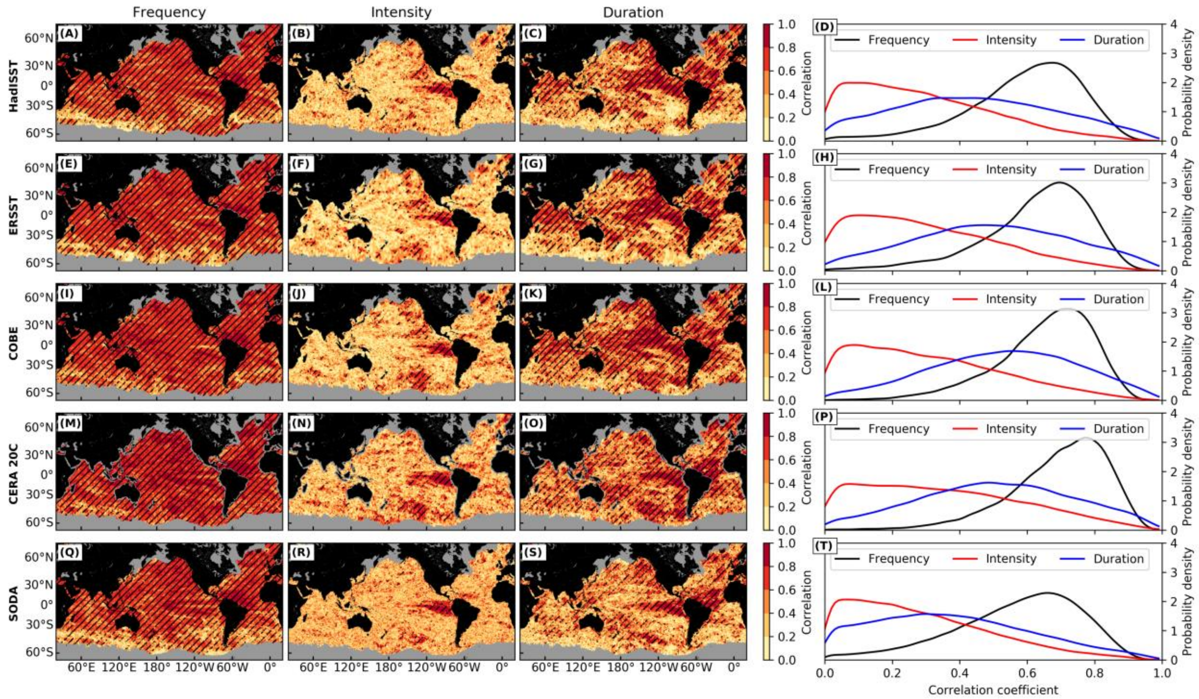

Supplementary Figure 7. Correlation coefficients for proxy models of NOAA OI SST marine heatwave properties based on the five monthly SST datasetsover 1982-2016. Heat maps show the spatial distribution of the correlation coefficients and line plots show the probability distributions of correlation values for all grid cells. Hatching indicates correlation coefficient is statistically significant at the 5% level; grey indicates missing or insufficient data.

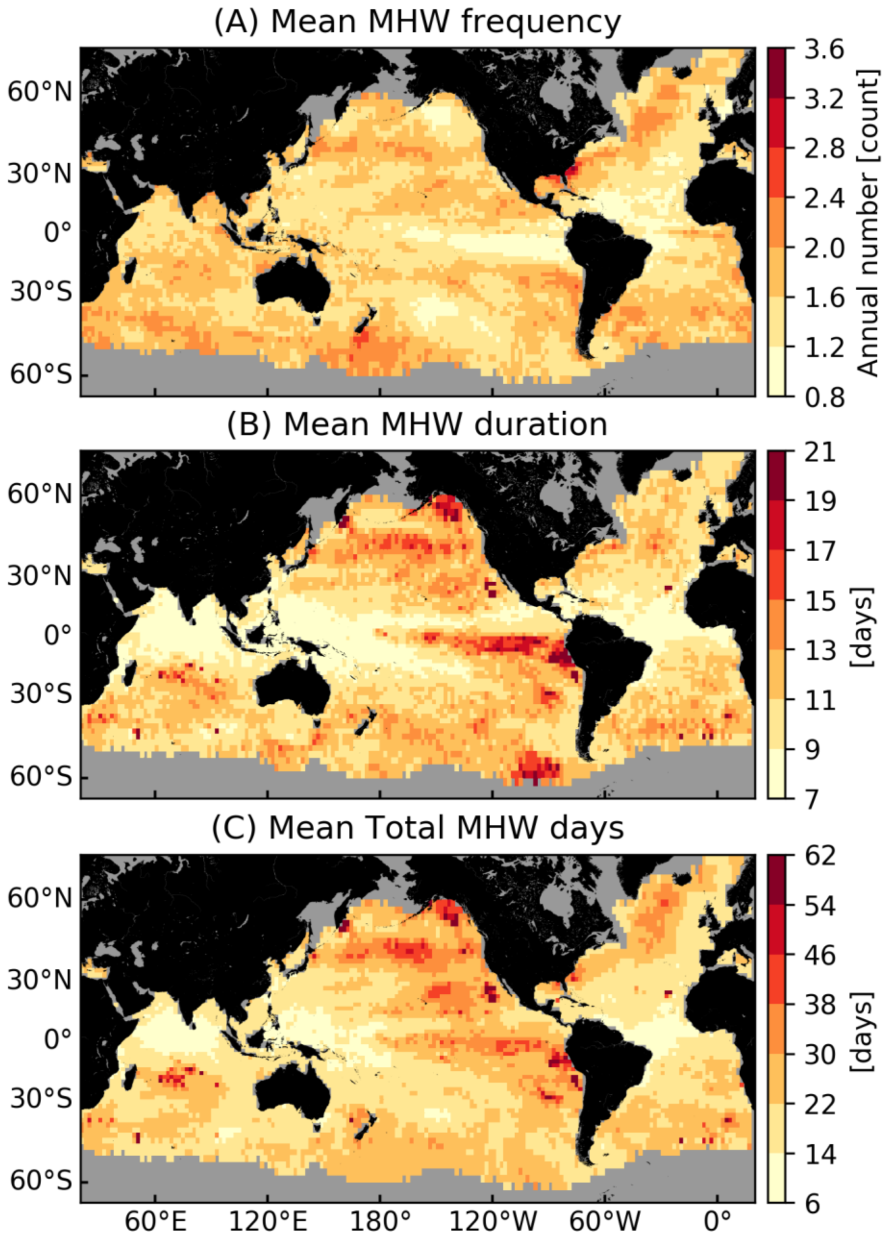

Supplementary Figure 8. Global marine heatwave properties derived from monthly gridded SST data. Mean of (A) annual marine heatwave count, (B) marine heatwave duration, and (C) total marine heatwave days from MHW proxies over 1900-2016. The metrics have been averaged across the five proxy datasets.

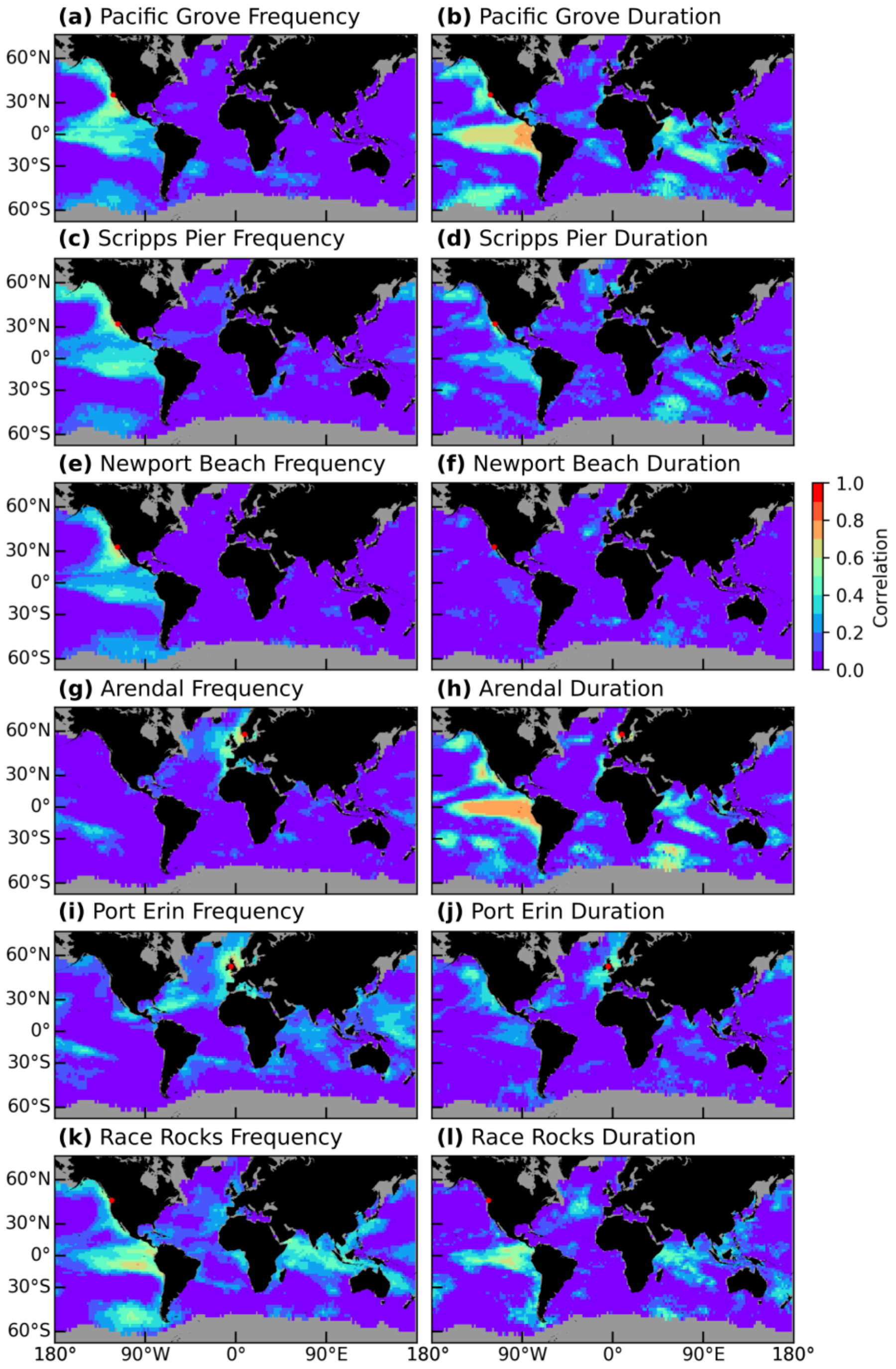

Supplementary Figure 9. Correlation of annual marine heatwave statistics at the long time series locations with the long proxies, globally. Correlations are shown for (A,C,E,G,I,K) frequency and (B,D,F,H,J,L) duration at each of the six stations. Station locations shown as red dots. Correlations were calculated for each proxy dataset separately, and then averaged across them at each grid point using the Fisher's *z*-transformation technique of ref. [5].

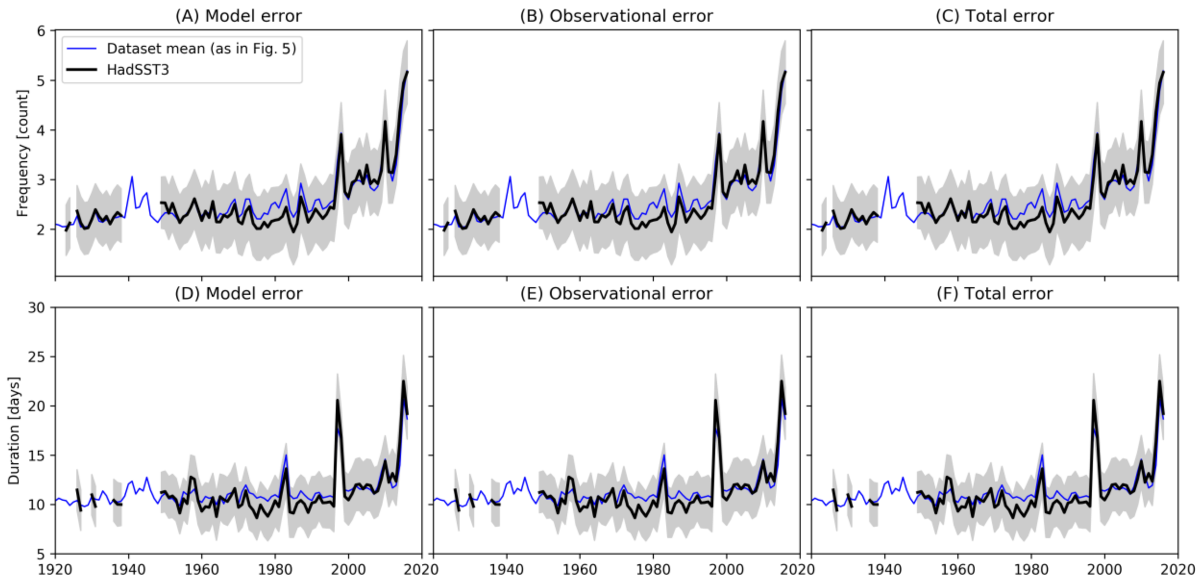

Supplementary Figure 10. Model and observational uncertainty estimates for the marine heatwave proxies. Globally averaged time series are shown for the HadSST3 proxies (thick black lines) and the dataset mean proxies (blue lines; as shown in Fig. 5, e.g. the mean of HadISST, ERSST, COBE, CERA20C and SODA proxies) of (A, B, C) marine heatwave frequency and (D, E, F) marine heatwave duration. Shaded regions indicate the 95% confidence intervals of the HadSST3 proxies derived from (A, D) uncertainties in the model fit, (B, E) sampling and measurement (observational) uncertainties and (C, F) the combination of model and observational (total) uncertainties.

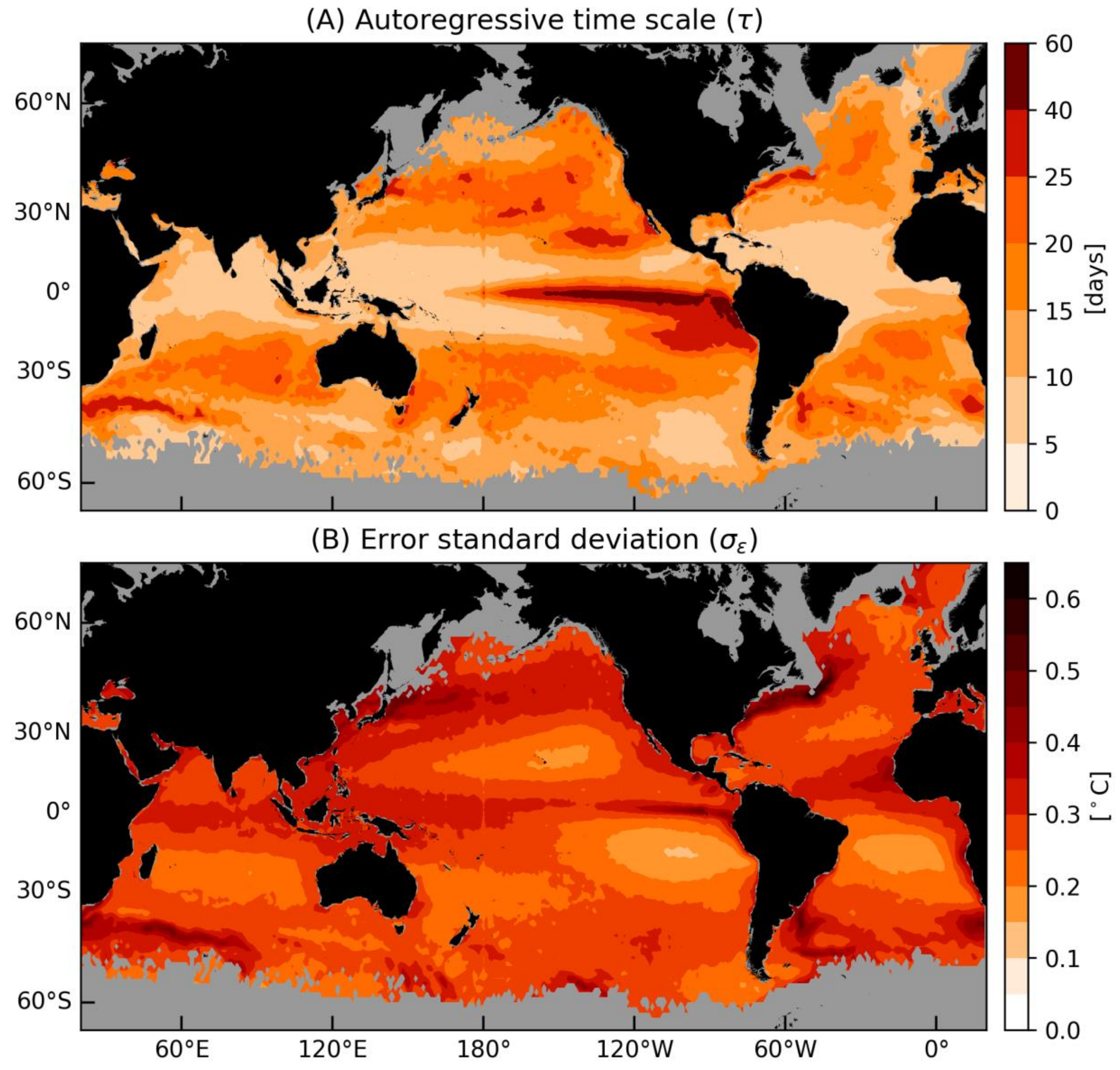

Supplementary Figure 11. Parameters for the stochastic climate model fit to NOAA OI SST. Shown are (A) the autoregressive time scale τ and (B) the standard deviation σ_ε of the white noise error forcing.
